# Recent Progress on PEDOT-Based Thermoelectric Materials

**DOI:** 10.3390/ma8020732

**Published:** 2015-02-16

**Authors:** Qingshuo Wei, Masakazu Mukaida, Kazuhiro Kirihara, Yasuhisa Naitoh, Takao Ishida

**Affiliations:** Nanosystem Research Institute, National Institute of Advanced Industrial Science and Technology, 1-2-1 Namiki, Tsukuba, Ibaraki 305-8564, Japan; E-Mails: qingshuo.wei@aist.go.jp (Q.W.); mskz.mukaida@aist.go.jp (M.M.); kz-kirihara@aist.go.jp (K.K.); ys-naitou@aist.go.jp (Y.N.)

**Keywords:** organic semiconductors, thermoelectrics, energy conversion, anisotropicity

## Abstract

The thermoelectric properties of poly(3,4-ethylenedioxythiophene) (PEDOT)-based materials have attracted attention recently because of their remarkable electrical conductivity, power factor, and figure of merit. In this review, we summarize recent efforts toward improving the thermoelectric properties of PEDOT-based materials. We also discuss thermoelectric measurement techniques and several unsolved problems with the PEDOT system such as the effect of water absorption from the air and the anisotropic thermoelectric properties. In the last part, we describe our work on improving the power output of thermoelectric modules by using PEDOT, and we outline the potential applications of polymer thermoelectric generators.

## 1. Introduction

Thermoelectric (TE) devices are promising candidates for harvesting waste heat and solar thermal energy [1]. Previous studies of TE materials have mainly investigated inorganic materials such as bismuth-telluride (Bi-Te) and metal oxides [2]. Most of these inorganic TE materials show their optimum performance at temperatures higher than 200 °C. However, there is a huge amount of waste heat, that is at temperatures lower than 150 °C [3]. At such low temperatures, the energy conversion efficiency is low because of the small achievable temperature difference suggested by the Carnot cycle. To harvest the huge amount of thermal energy available at low temperatures, ideal materials should be cheap to produce, flexible, and suitable for large-area fabrication. Organic semiconductors meet these requirements.

Studies of the TE properties of organic semiconductors, such as the Seebeck effect, have been conducted previously. The Seebeck effect has been used as a physical parameter to determine the carrier type and relative carrier concentration in organic conducting materials such as polyaniline, polypyrrole, and polythiophene [4,5,6]. Recent studies of organic electronics, including organic solar cells and transistors, have improved the physical and chemical properties of organic conducting polymers [7]. They can now be tuned over a wide range, which may make them suitable for other energy conversion technologies such as TE devices. Poly(3,4-ethylenedioxythiophene):poly(styrenesulfonate) (PEDOT:PSS) is the most studied conducting polymer because it shows high electrical conductivity when a suitable second solvent, such as ethylene glycol (EG) or dimethyl sulfoxide (DMSO), is added to an aqueous dispersion [8,9,10]. Furthermore, PEDOT:PSS is commercially available on a large scale. *In situ* synthesized PEDOT:tosylate (tos) has also been widely studied because of its easy fabrication and good electrical performance [11,12,13,14,15].

In this review, we report recent progress in TE studies concerning the PEDOT system, one of the most promising organic materials for large-area TE devices designed to function around room temperature. We start from the origin of the high electrical conductivity of the PEDOT system. Then, we summarize the recent TE performance of PEDOT systems. Finally, we present our PEDOT:PSS module fabrication and outline the potential applications of organic TE devices.

## 2. Basis of the PEDOT System and the Origin of Its High Electrical Conductivity

Elschner *et al.* from H.C. Starck Clevios have published a comprehensive book covering the history of PEDOT and technical details of its synthesis [16]. In this paper, we mainly describe recent progress in improving the electrical conductivity of this system.

**Figure 1:**
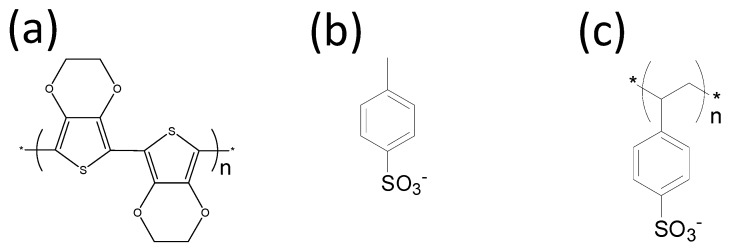
Molecular structures of (**a**) poly(3,4-ethylenedioxythiophene) (PEDOT), (**b**) tosylate (tos), and (**c**) poly(styrenesulfonate) (PSS).

Figure 1a shows the chemical structure of PEDOT. The synthesis of PEDOT was first reported in 1988 [17]. It was designed and synthesized as a conducting polymer that is stable toward moisture and oxygen; existing conducting polymers, such as polyacetylene, polyaniline, and polypyrrole, exhibited low atmospheric stability, which limited their applications. The most common synthetic route to PEDOT is oxidative polymerization from 3,4-ethylenedioxythiophene (EDOT). Conductive PEDOT carries positive charges; therefore, counterions, such as chloride (Cl^−^), perchlorate (ClO_4_^−^), tos (Figure 1b), and polystyrene sulfonate (PSS; Figure 1c), were present in all oxidized forms of PEDOT for charge balancing. Normally, these counterions are called dopants, although these counterions cannot oxidize PEDOT, and a simple mixture of PEDOT and the counterions does not change the doping state. P-type doping in organic semiconductors is an oxidation reaction, and thus the actual dopants are oxidants, such as Fe^3+^, that are removed after synthesis.

Generally, conductive PEDOT films can be formed by two methods. The first method is the synthesis of the PEDOT during film formation, which is called *in situ* polymerization. A typical example is PEDOT:tos, which is synthesized using Fe(tos)_3_ as an oxidant. The highest electrical conductivity reported for a thin film of PEDOT:tos was 4300 S/cm [18]. Recently, single-crystalline PEDOT nanowires were synthesized under geometrically confined conditions using patterned substrates, and a high electrical conductivity of 8797 S/cm was achieved [19]. Metallic behavior of the *in situ* polymerized PEDOT films was also reported [20].

**Figure 2:**
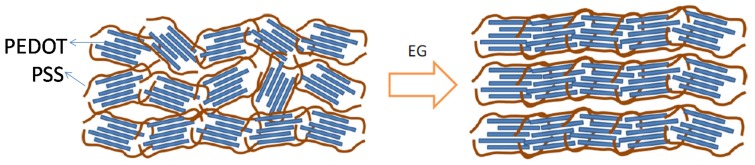
Morphological change in the PEDOT:PSS films caused by the addition of ethylene glycol (EG). Reproduced with permission from [22]. Copyright 2013 Wiley−VCH Verlag GmbH & Co.

The second method of forming conductive PEDOT films is coating from a stable PEDOT dispersion. This approach is limited to using PEDOT:PSS because it is the only stable PEDOT dispersion commercially available on a large scale [16]. There are different grades of PEDOT:PSS depending on the application. Grades Al4083 and PH1000 from Heraeus (Hanau, Germany) are commonly used. Al4083 was widely used as a hole-transporting layer in organic solar cells and organic light-emitting diodes. PH1000 exhibits the highest electrical conductivity and could be used as an alternative to indium tin oxide as a transparent conducting substrate. For solution-processed PEDOT:PSS films, the addition of a high boiling point solvent, such as EG or DMSO, can increase the electrical conductivity of the film [8,9,10]. PH1000 achieved an electrical conductivity of up to 1000 S/cm. Takano *et al.* used synchrotron X-ray diffraction to observe the formation of PEDOT nanocrystals after cosolvents were added [21]. We focused on the film structure and reported why the addition of EG can significantly increase the electrical conductivity of PH1000 based on morphological studies using grazing-incidence wide-angle X-ray diffraction (GIWAXD) and grazing-incidence small-angle X-ray scattering (GISAXS) [22]. GIWAXD indicated that the average crystal size was increased by adding EG to the solution. PEDOT showed face-on packing, and the π-conjugated planes of the PEDOT crystal were more vertical with respect to the substrate. GISAXS suggested that the addition of EG increased the order of the PEDOT nanocrystals; these nanocrystals form a layered structure. The results showed that the cosolvent helps to improve the crystallinity and ordering of the PEDOT nanocrystals in solid films (Figure 2). The carrier mobility and carrier density in highly conductive PEDOT are very important parameters for improving the material performance; however, these parameters have remained relatively unexplored in solid PEDOT films. This is probably because of the high carrier density in PEDOT and mobile ions such as H^+^ in the film. As the Hall voltage is inversely proportional to the carrier density, measuring the mobility of the conducting polymer using the Hall Effect is difficult. The carrier density and mobility have been estimated using different techniques [23,24,25,26,27]. For example, Yamashita *et al.* demonstrated that terahertz and IR–UV (infrared-ultraviolet) spectroscopy can be used to determine the carrier mobility by fitting the results with theoretical models [27]. We studied the carrier transport properties by using ion-gel transistors combined with *in situ* UV–vis–NIR (near infrared) spectroscopy, because ion gel shows a large specific capacitance that could introduce a higher density of charge carriers in the transistors [22]. The carrier mobility extracted from thin film transistors of highly conductive PH1000 films with EG addition was 1.7 cm^2^/Vs and the carrier density was in the order of 10^21^ cm^−3^. For the film without EG addition, the carrier mobility showed a much lower value of 0.045 cm^2^/Vs and the carrier density was on the order of 10^20^ cm^−3^. These results provide direct evidence that the improvement in electric conductivity in PEDOT:PSS following the addition of EG is mainly caused by improving the carrier mobility, and that it also increases the calculated carrier density. It is important to note that the doping of p-type semiconductors is an oxidation reaction and that oxidizing agents, such as I_2_ or Fe^3+^, are necessary for doping. A cosolvent like EG cannot oxidize PEDOT, although oxygen in the air could act as a dopant. PEDOT with a highly ordered structure could react with oxygen more easily during annealing, which would increase the carrier density. Recently, it was reported that treating the PH1000 films in strong acid, such as a sulfuric acid, produced a very high electrical conductivity of 4380 S/cm [28,29]. The reasons for the improved carrier mobility and carrier density were investigated.

## 3. TE Measurements

The energy conversion efficiency is an important parameter for evaluating the energy conversion performance of TE devices. The thermodynamic limit of the energy conversion efficiency is determined by reversible Carnot cycles. For TE devices, the device efficiency cannot be easily extracted like solar cells [30]. In the simplest case, the energy conversion efficiency is related to the materials’ dimensionless figure of merit (*ZT*); therefore, *ZT* is crucial for selecting and optimizing TE materials. *ZT* is defined as *ZT* = *S*^2^σ*T*/κ, where *S* is the Seebeck coefficient, σ is the electrical conductivity, *T* is the absolute temperature, and κ is the thermal conductivity. The power factor (*PF* = *S*^2^σ) is used to evaluate the power generation of a material at a certain temperature difference. 

**Figure 3:**
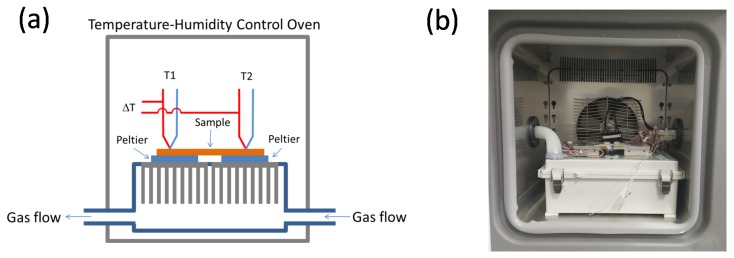
(**a**) Schematic and (**b**) photographic images of the Seebeck measurement setup in high-humidity conditions. Reproduced with permission from [31]. Copyright 2014 Japan Society of Applied Physics.

The Seebeck coefficient, *S*, is measured by dividing the voltage difference, Δ*V*, by its corresponding temperature difference, Δ*T*. There are several commercial measurement systems available. For example, the ZEM series from ULVAC Technologies (Chigasaki, Japan) can be used to measure the Seebeck coefficient over a very large temperature range, and the SB-100 system from MMR Technologies (Mountain View, CA, USA) is suitable for small samples on sub-millimeter scales. For conducting polymers, custom-made equipment is preferred because of the low conductance of thin films and because the composition of the films could change at different measurement environments. Our custom-made Seebeck coefficient measurement system is shown in Figure 3. The sample holder with two Peltier units was placed in a temperature/humidity-controlled oven. The temperatures of the Peltier units and the oven were controlled independently. The thermocouples are attached to the sample directly with a point contact. The temperature difference (Δ*T*) of the sample and the electromotive force (Δ*V*) were measured simultaneously by probing the pair of electrodes with a digital multimeter. The slope of the plot of Δ*V* *versus* Δ*T* gave *S*.

Another approach to measuring the Seebeck coefficient is through fabricating evaporated metal electrodes on the sample through a shadow mask, and probing the voltage and temperature between the two electrodes. This approach is vital for studies of organic thermoelectric materials because many materials may not form ohmic contact with the thermocouples by simple attachment. Contact between evaporated metal electrodes and organic films is generally better and the work function of the electrode can be tuned to match the energy levels of the organic film. However, the contact geometry is very important for accurate measurements [32]. To calibrate the Seebeck coefficient measurement system, pure Ni foil is a good reference because of its relatively large Seebeck coefficient and specific temperature dependence [33].

The electrical conductivity, σ, is measured by using the four-probe method to exclude the effect of contact resistance. Linear and van der Pauw geometry apply in thin conducting polymer films. The flash analysis method and differential three-omega (3ω) methods are standard methods for measuring the thermal conductivity [34,35]. The flash analysis method requires a dense freestanding film with a thickness greater than tens of micrometers. The differential 3ω method works for a thin film as thin as tens of nanometers [36].

Organic TE materials are often characterized by making a film and measuring the in-plane electrical conductivity, in-plane Seebeck coefficient, and through-plane thermal conductivity. This is because it is difficult to make a dense block on a millimeter scale using a solution-based process. The preferred molecular orientation during film formation means that the electrical conductivity, Seebeck coefficient, and thermal conductivity can be anisotropic. In other words, the calculation of *ZT* for organic thermoelectric materials may not be as straightforward as for their inorganic counterparts, and it could vary greatly. To estimate the TE properties of organic films reliably, measuring these parameters in both directions is critically important for understanding the thermal and carrier transport mechanism in conducting polymers and for improving their TE performance. We describe our evaluation of TE properties in the in-plane and though-plane directions later [37].

## 4. TE Performance of PEDOT Systems

Because it is difficult to summarize the TE properties of PEDOT films in the in-plane and through-plane directions separately at this stage, the anistropicity is not considered here. The morphology of the organic films could easily be affected by the preparation process; therefore, the anisotropicity or isotropicity could vary depending on fabrication conditions.

In early work, the reported power factors for organic TE materials were less than 10 μW/(m K^2^) [4,10,38,39,40,41,42,43,44]. After 2010, several groups reported high power factors and excellent *ZT* for materials based on PEDOT films. Katz and co-workers first reported a promising power factor of 47 μW/(m K^2^) for commercial PEDOT:PSS (PH1000) with a higher electrical conductivity of 900 S/cm, with a Seebeck coefficient of 20 μV/K [45].

**Figure 4:**
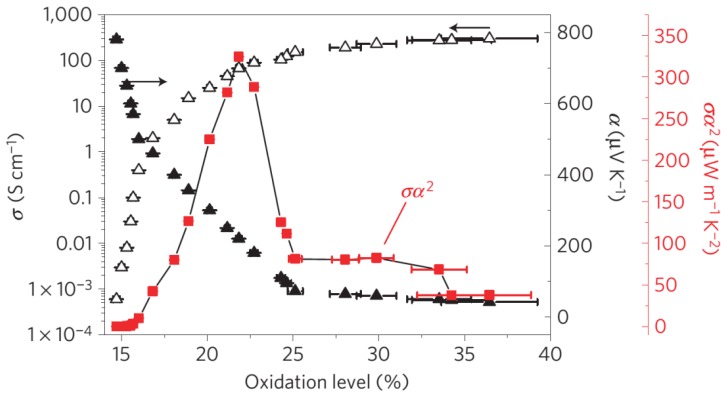
Seebeck coefficient (filled triangles), electrical conductivity (open triangles) and corresponding power factor (red squares) *versus* oxidation level of the PEDOT/tos films. Reproduced with permission from [46]. Copyright 2011 Nature Publishing Group.

**Figure 5:**
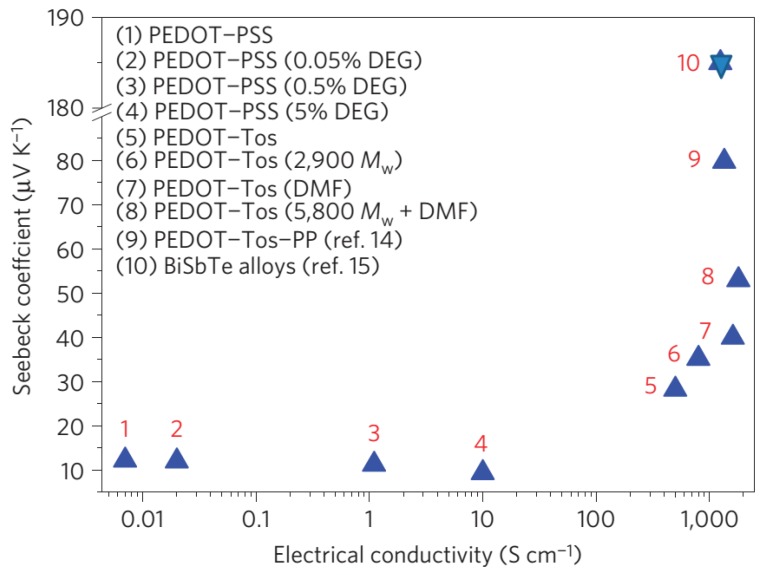
Seebeck coefficient *versus* electrical conductivity of various PEDOT derivatives including pristine PEDOT/PSS and diethylene glycol (DEG)-containing samples, chemically polymerized PEDOT/Tos as well as three VPP PEDOT/Tos samples with triblock copolymers PEG–PPG–PEG with different Mw. Reproduced with permission from [48]. Copyright 2014 Nature Publishing Group. (PEG = polyethylene glycol, PPG = polypropylene glycol).

**Figure 6:**
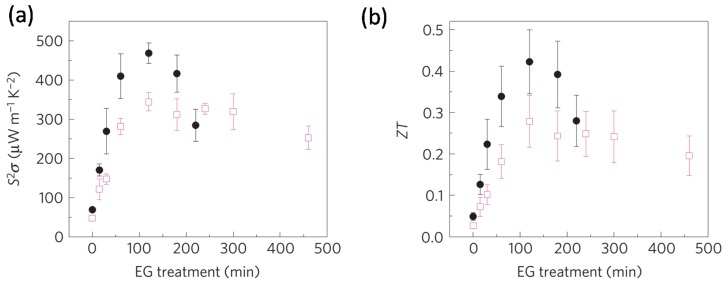
(**a**) Thermoelectric power factors and (**b**) thermoelectric figure-of-merit at 297 K in EG-mixed (open squares) and dimethyl sulfoxide (DMSO)-mixed (closed circles) PEDOT:PSS measured during the EG treatment (dedoping) process. Reproduced with permission from [49]. Copyright 2013 Nature Publishing Group.

In 2011, Crispin and colleagues reported that de-doping *in situ* polymerized highly conductive PEDOT:tos with tetrakis(dimethylamino)-ethylene (TDAE) can generate a remarkable power factor larger than 300 μW/(m K^2^) (as shown in Figure 4) and *ZT* of 0.25, which is mainly attributed to a high Seebeck coefficient (>200 μV/K) [46]. They also demonstrated the fabrication of π-type thermoelectric generators by using the de-doped PEDOT:tos films. In 2013, Kim and co-workers reported a different approach to controlling the doping levels of PEDOT:tos films, by using electrochemistry. The pristine *in situ* synthesized PEDOT films gave a power factor of 862.9 μW/(m K^2^), and slightly de-doped films showed a very high power factor of 1290 μW/(m K^2^) [47]. To explain the improved TE performance, Crispin and co-workers synthesized a series of PEDOT:tos and studied the relationship between the electrical conductivity and Seebeck coefficient. They observed an increase in Seebeck coefficient with the electrical conductivity (as shown in Figure 5), which indicates that PEDOT:tos could have semi-metallic properties [48]. Because semi-metallic polymers have zero bandgap and a very low density of states around the Fermi level, they typically exhibit a higher Seebeck coefficient and lower thermal conductivities compared with metallic polymers, and are thus suitable for TE applications. Crispin and co-workers suggested that PEDOT:tos can form a semi-metal because the carriers are only bipolarons [48]. In 2013, Pipe and colleagues reported a higher power factor of 469 μW/(m K^2^) and a *ZT* value of 0.42 at room temperature by mixing DMSO to commercial PEDOT:PSS [49]. The highest *ZT* was achieved after the EG treatment process for about 2 h as shown in Figure 6. They suggested that the improved TE performance can be attributed to increased carrier mobility and decreased carrier density arising from the selective removal of PSS anions. Massonnet and co-workers studied the relationship between the redox potential of the reducing agents and the Seebeck coefficient of the PEDOT:PSS film, and how the redox potential could be used to control the carrier density [50]. Furthermore, other groups also reported high power factors of over 100 μW/(m K^2^) on PEDOT films through chemical reduction with different chemicals (Table 1) [51,52,53].

**Figure 7:**
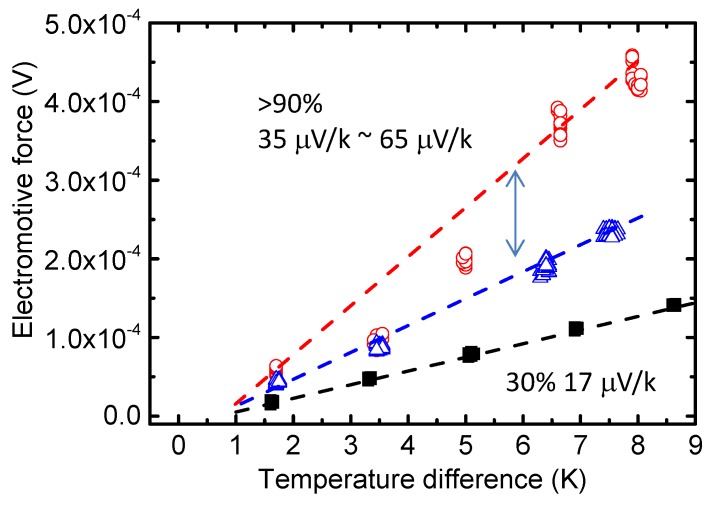
Seebeck coefficient measurements of PEDOT:PSS films at different humidity levels. Closed circles represent Seebeck coefficient measurements at a humidity of 30% and open circles represent Seebeck coefficient measurements at a humidity larger than 90%. Reproduced with permission from [31]. Copyright 2014 Japan Society of Applied Physics.

However, the reported Seebeck coefficients or *ZT* values vary widely, even using commercial materials, suggesting that there are still aspects of the PEDOT system that are not well understood. Recently, we reported the effect of the measurement environment on the apparent Seebeck coefficient of PEDOT:PSS films [31]. PEDOT:PSS readily absorbs water vapor from air, and the weight percent of water in PEDOT:PSS is appreciable. Therefore, the effects of water on the TE properties of PEDOT:PSS should be considered. We confirmed an increase in the thermoelectric power factor of PEDOT:PSS from 23–355 μW/(m K^2^) in extremely high-humidity conditions (Figure 7). This increase was caused mainly by an increase in the apparent Seebeck coefficient, which could be related to morphological changes after water absorption or the electrochemical reactions of PEDOT in air [54,55,56]. Our results demonstrate the positive effect of water in the PEDOT:PSS film and highlight the need for well controlled measurement conditions, particularly humidity, in evaluating the performance of conducting organic materials.

**Table 1:** Thermoelectric (TE) properties of poly(3,4-ethylenedioxythiophene) (PEDOT)-based materials. (tos = tosylate, and PSS = poly(styrenesulfonate)).

Materials	S (μV/K)	PF (μW/m K^2^)	*ZT*	Ref.
PEDOT:PSS	22	47	0.1	[45]
PEDOT:tos (dedoped)	200	324	0.25	[46]
PEDOT:PSS	73	469	0.42	[49]
PEDOT:tos	~85	1290	-	[47]
PEDOT:tos	55	453	-	[48]
PEDOT:BTFMSI	~40	147	0.22	[53]
PEDOT:PSS (dedoped)	~50	112	0.093	[51]
PEDOT:PSS (dedoped)	43	116	0.2	[52]
PEDOT:PSS	65	355	~0.3	[31]

Hybrid materials using conducting polymers and inorganic TE materials have been studied. A recent review article by Pentzer *et al.* covering recently reported polymer composites that have potential application as thermoelectric generators, is recommended [57]. There are several expected effects of using hybrid materials other than conducting polymers or inorganic TE materials, such as improved electrical conductivity because of polymer ordering, and an increased Seebeck coefficient through interface scattering and reduced thermal conductivity compared with inorganic TE materials. There are numerous studies of PEDOT/inorganic hybrid composites such as PEDOT:PSS/carbon nanotubes (CNTs) [58,59], PEDOT:PSS/Te nanorods [60,61], PEDOT:PSS/Ca_3_Co_4_O_9_ [62], PEDOT:PSS/PbTe [63], PEDOT:PSS/Bi_2_Te_3_ [45], PEDOT:PSS/Au nanoparticles [64,65], and PEDOT:PSS/Ge (Table 2) [66]. PEDOT:PSS/CNTs showed good thermoelectric performance because PEDOT:PSS modifies the junctions between CNTs, suppressing phonon transport while maintaining the good electronic properties. The thermal conductivity of the composites was maintained within the range of typical organic materials of 0.2–0.4 W/(m K) and a high power factor of 160 μW/(m K^2^) was achieved [58]. Multiwall CNTs improved the power factor further to 500 μW/(m K^2^) [59]. See and co-workers synthesized a water-soluble composite of Te/PEDOT:PSS with a core-shell structure. The solution-processed nanocrystal films show an improved power factor of 70.9 μW/(m K)^2^. Because the film retains a low thermal conductivity, a high *ZT* value of 0.1 was achieved [60]. Very recently, Park and co-workers fabricated PEDOT:PSS/Ge composite films by ball-milling under nitrogen. A high power factor of 165 μW/(m K^2^) was achieved because of the high Seebeck coefficient, and this film also retains a low thermal conductivity, resulting in a *ZT* of 0.1 at room temperature [66]. In hybrid materials, both the electrical conductivity and Seebeck coefficient of the films could decrease if a homogeneous mixture of the materials cannot be obtained. This is because carriers flow through each material independently.

**Table 2:** TE properties of PEDOT-based hybrid films.

Materials	S (μV/K)	PF (μW/m K^2^)	*ZT*	Ref.
PEDOT:PSS/SWCNT	30	25	0.02	[59]
PEDOT:PSS/MWCNT	70	500	-	[60]
PEDOT:PSS/Bi_2_Te_3_	60	130	0.1	[45]
PEDOT:PSS/Te	163	70.9	0.1	[61]
PEDOT:PSS/Au NPs	26.5	51.2	~0.1	[65]
PEDOT:PSS/Au nanorods	12	30	-	[66]
PEDOT:PSS/Ge	~50	165	0.1	[67]

The thermal stability and flexibility of PEDOT films are important for TE applications. Hokazono *et al.* investigated the thermal stability and flexibility of PEDOT:PSS films [67]. The thermal stability was evaluated by measuring the Seebeck coefficient and electrical conductivity for 30 heating and cooling cycles at 330–380 K. They also examined the durability of the PEDOT:PSS film by heating at 353 K in air for 4000 h. The Seebeck coefficient and electrical conductivity of PEDOT:PSS film were nearly constant during the heating cycle treatments. For the durability test, the Seebeck coefficient of the PEDOT:PSS film was unchanged before and after repeated bending for 10,000 times at a curvature radius of 10 mm. However, the electrical conductivity gradually decreased with increasing heating time. The data shows that PEDOT:PSS films are flexible and mechanically tough and also have relatively good thermal stability in air up to 3600 h.

## 5. Anisotropic TE Properties of PEDOT:PSS Films

Organic TE materials are often characterized by the in-plane electrical conductivity, in-plane Seebeck coefficient, and through-plane thermal conductivity of a thin film, because it is difficult to obtain the through-plane electrical conductivity, through-plane Seebeck coefficient, and in-plane thermal conductivity of a thin conducting polymer film. As many organic semiconductors form an anisotropic film, we can expect that these anisotropic films will show anisotropic TE properties.

Although measuring the through-plane four-probe electrical conductivity, through-plane Seebeck coefficient, and in-plane thermal conductivity is important for understanding the thermal transport and carrier transport mechanism in conducting polymers to improve their TE performance, it is challenging. Furthermore, the TE properties in both directions also affect thermoelectric device design. Recently, we reported an approach for studying the TE properties in both directions by using the benchmark conducting polymer PEDOT/PSS [37].

**Figure 8:**
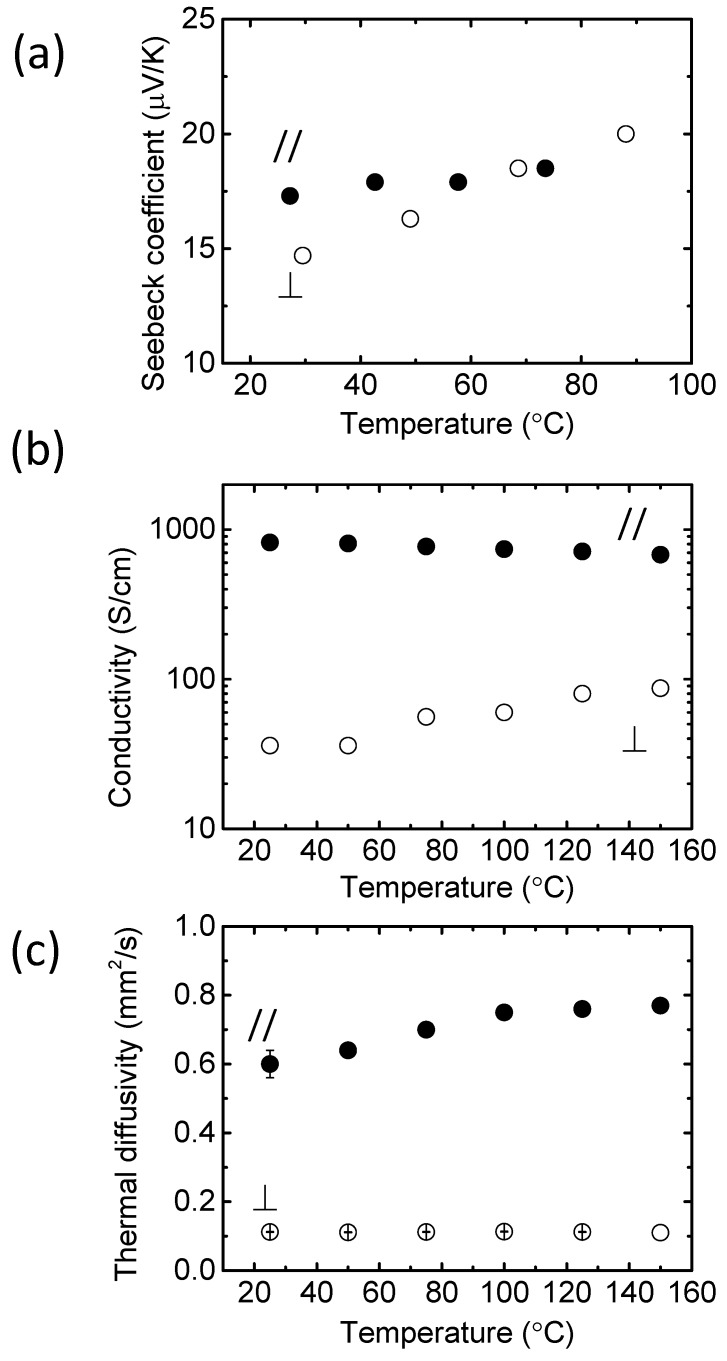
(**a**) Seebeck coefficient, (**b**) electrical conductivity, and (**c**) thermal diffusivity of the PEDOT/PSS films in the in-plane (closed circles) and through-plane (open circles) directions, plotted as a function of temperature. Reproduced with permission from [37]. Copyright 2014 American Chemical Society.

We measured the through-plane Seebeck coefficient of PEDOT/PSS by using a homemade Seebeck coefficient measurement setup. Figure 8a shows the through-plane Seebeck coefficient measurements for the free standing PEDOT/PSS film. The Seebeck coefficient in the through-plane direction was 15 μV/K at room temperature, which is close to the in-plane value.

We observed higher anisotropy in the electrical conductivity and thermal diffusivity. As shown in Figure 8b,c, the in-plane electrical conductivity of the PEDOT/PSS films was measured as 820 S/cm, which is identical to the previously reported value, suggesting this thick film had an ordered structure similar to the thin films. However, the through-plane conductivity showed a lower value of 36 S/cm. The anisotropic electronic conductivity was attributed to the morphology of the PEDOT/PSS films. We have also shown that highly conductive PEDOT/PSS films have a layered structure, and PSS may isolate the PEDOT nanocrystals in the through-plane direction [22]. We used flash analysis to measure the in-plane thermal diffusivity, using large-area, freestanding PEDOT/PSS films. We calculated the thermal conductivity by using the thermal diffusivities. The estimated thermal conductivities were 0.15 W/(m K) in the through-plane direction and 0.84 W/(m K) in the in-plane direction, suggesting that the thermal conductivity of the ordered PEDOT/PSS films was also anisotropic.

We calculated *ZT* at room temperature; it was 8.4 × 10^−3^ in the in-plane direction and 1.6 × 10^−3^ in the through-plane direction. The *ZT* calculated by using the in-plane electrical conductivity, in-plane Seebeck coefficient, and through-plane thermal conductivity gave an overestimated value of 0.05.

To improve the TE performance of PEDOT, one possible approach is to reduce the in-plane thermal conductivity by using a smaller dopant. PEDOT/tos, in which the PSS was replaced with smaller anions, showed almost isotropic thermal conductivity [46]. Therefore, small dopants and controlling the crystal orientation may be effective strategies for optimizing the TE performance.

## 6. TE Module Designing and Fabrication

**Figure 9:**
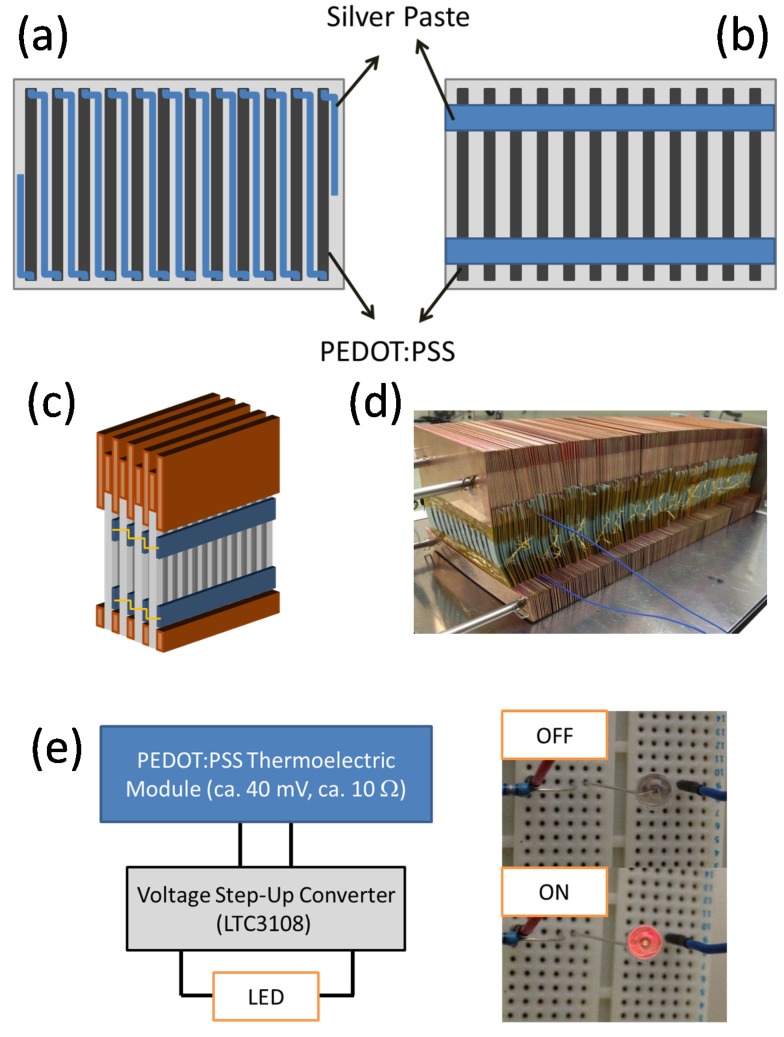
Schematic representation of the (**a**) series and (**b**) parallel PEDOT:PSS array. (**c**) Schematic and (**d**) photograph of the PEDOT:PSS modules sandwiched between copper plates. (**e**) Schematic of the circuit for powering an LED and photograph of the LED driven by the PEDOT:PSS thermoelectric module. Reproduced with permission from [68]. Copyright 2014 The Royal Society of Chemistry.

Conventional inorganic TE modules have π-type structures, which combine both p-type and n-type materials to convert thermal energy to electrical energy efficiently. In principle, organic conducting polymers can be doped to form both p-type and n-type materials; however, n-type doping is difficult. Studies using only p-type conducting polymers to make TE modules are still limited at the moment. Furthermore, in the conventional device configuration, the TE performance is determined by the through-plane electrical conductivity and the through-plane Seebeck coefficient. It is difficult to make dense blocks of conducting polymers on the centimeter scale; therefore, techniques to obtain larger temperature differences are required to fabricate an organic TE module with PEDOT films. We have studied devices with a fin structure fabricated from screen-printed PEDOT:PSS on paper to achieve a larger temperature difference (Figure 9a,b) [68]. A PEDOT:PSS layer 2.5 mm × 40 mm and about 20 μm thick was screen-printed on a 300-μm-thick piece of paper. Eleven PEDOT:PSS arrays were printed on one piece of paper, and the distance between the arrays was 8 mm. Silver paste was screen-printed on the PEDOT:PSS arrays to create connections. Silver paste was used because of its high conductivity, low sintering temperature, and good wettability on PEDOT:PSS. The PEDOT/silver paste arrays were sandwiched between copper plates (Figure 9c,d), and they were connected either in series or in parallel with metal wires.

A temperature gradient occurs between the top and bottom of the paper (Figure 9a). There are two reasons why we used paper as the module substrate. First, paper shows better thermal stability than polymer substrates, such as polyethylene terephthalate and polyethylene naphthalate, which have a glass transition temperature lower than 150 °C. Secondly, because the PEDOT:PSS ink is water-based, it should have better wettability on paper than on typical polymer substrates such as PET films. Jiang *et al.* also reported that paper is an effective substrate for preparing high-performance flexible TE materials [69]. Our large-area devices gave a power output of over 50 μW at Δ*T* of about 100 K, which provided sufficient power to illuminate an LED (Figure 9e).

**Figure 10:**
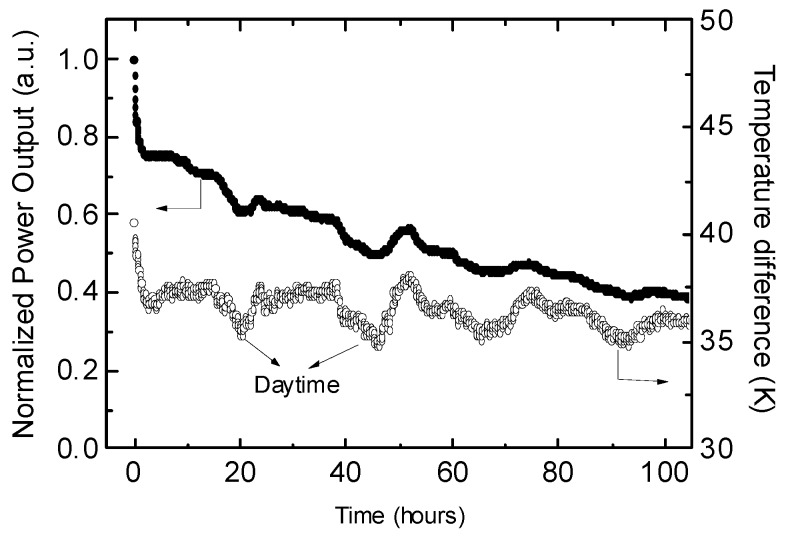
Power output of module and temperature difference as a function of the working time. Reproduced with permission from [68]. Copyright 2014 The Royal Society of Chemistry.

**Figure 11:**
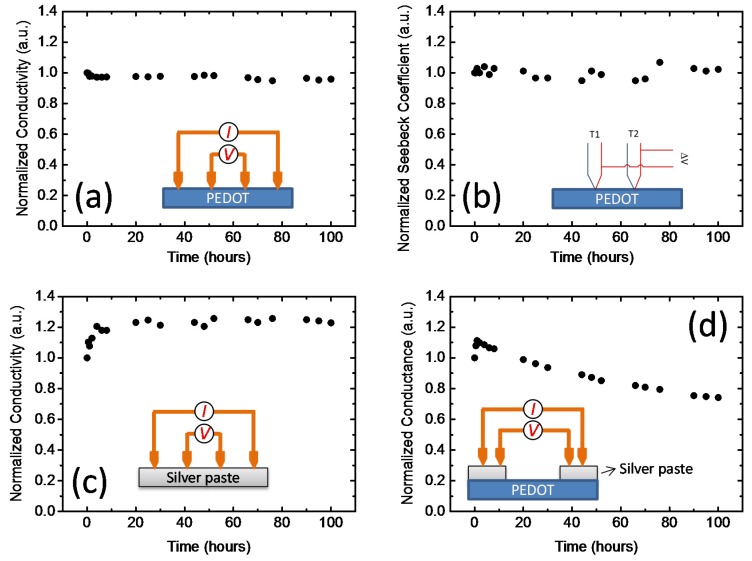
(**a**) Electrical conductivity and (**b**) Seebeck coefficient of PEDOT:PSS, and (**c**) electrical conductivity of silver paste and (**d**) conductance of silver paste/PEDOT:PSS/silver paste as a function of time at 80 °C. Reproduced with permission from [68]. Copyright 2014 The Royal Society of Chemistry.

Although PEDOT:PSS showed remarkable thermal stability in air, the performance of our fabricated TE module decreased over time (Figure 10). To explain the decrease in the TE device performance during continuous operation for 100 h, we carried out further thermal electrical stability measurements for each material. Figure 11a,b show the four probe electrical conductivity and Seebeck coefficient of PEDOT:PSS as a function of time at 80 °C. Both the electrical conductivity and Seebeck coefficient were very stable over time. Figure 11c shows the four-probe electrical conductivity of the Ag paste as a function of time. During the first 10 h, the electrical conductivity increased about 20%, and then it remained almost constant. The increase in the electric conductivity may be caused by the evaporation of the solvent incorporated in the Ag paste and the aggregation of the Ag nanoparticles in the ink. These results indicate that PEDOT:PSS and the Ag paste are sufficiently thermally stable. Figure 11d shows the conductance change of the Ag paste/PEDOT:PSS/Ag paste sandwiched device. The conductance decreased to about 70% of its initial value after 100 h heating, a tendency that was similar to the decrease in the power output of the TE modules. If both PEDOT:PSS and Ag paste are stable, the decrease in conductance of the sandwiched structure must be caused by an unstable interface. To confirm the changes at the Ag/PEDOT:PSS interface, we carried out X-ray photoelectron spectroscopy (XPS) measurements. XPS showed that the PSS/PEDOT ratio increased to 3.5 after the stability test for 100 h, which is higher than the PSS/PEDOT ratio in solution and PEDOT:PSS/air interface (2.5). Because PSS is an insulator, the segregation of PSS increased the contact resistance between PEDOT:PSS and Ag paste. This suggests that the interface between the conducting polymer and electrode is important for improving the device stability.

Although our fin structure TE modules could harvest waste thermal energy in practical situations, the size of the module must be reduced, for example by reducing the polymer thickness and developing freestanding films. We are currently fabricating thinner TE modules using PEDOT films with a higher power density.

## 7. Future Outlook

Application-orientated research into organic TE devices and materials has just started, and recent efforts have shown that high *ZT* and power factors could be achieved by using conducting polymers. In addition to the material properties, improving the power output of the flexible devices is also important.

To increase the power output of TE modules, the temperature difference must be increased by increasing the heat radiation efficiency from the TE module or by another suitable technique. For example, for inorganic TE devices, Sakai *et al.* recently reported the “trade-off-free” interdependence between thermal conductivity and electrical conductivity in an inorganic Bi_0.5_Sb_1.5_Te_3_ multilayer [70]. They substantially increased the TE power based on the off-diagonal thermoelectric effect, which induced a transverse electrical current in response to the vertical thermal current. They obtained a higher output power of 2.5 kW/m^2^ with a Δ*T* of 85 K at a temperature of less than 100 °C [70]. If a similar approach can be used for conducting polymer TE modules, we can expect to fabricate organic TE devices with higher power outputs.

Energy harvesting, which targets very low power densities, is a possible application of organic thermoelectric devices [71]. Recent progress in semiconductor technology has decreased the power consumption of large-scale integration (LSI) devices. The combination of organic TE devices and low-power LSI devices is promising for energy harvesting [71]. Furthermore, organic TE devices could also be used in sensors, such as temperature sensors or monitors with large-area conducting polymer electrodes.

## 8. Conclusions

In this review, we report recent advances in improving the thermoelectric properties of PEDOT-based materials. Controlling the carrier concentration improves the material performance. The highest reported *ZT* value is 0.42 for PEDOT:PSS. These pioneering studies have opened new possibilities for the application of organic semiconductors. The improved material properties mean that the organic thermoelectric modules have a power output that can be used to power practical devices. This demonstrates that it is possible to convert thermal energy to electricity with organic semiconductors. For further improvement, the anisotropicity and stability at the interface should be considered.
